# Co-expressing LRP6 With Anti-CD19 CAR-T Cells for Improved Therapeutic Effect Against B-ALL

**DOI:** 10.3389/fonc.2020.01346

**Published:** 2020-09-15

**Authors:** Ping He, Zhongqiu Tan, Zhongheng Wei, Cheng-Liang Wan, Shan-Shan Yang

**Affiliations:** ^1^School of Laboratory Medicine, Youjiang Medical University for Nationalities, Guangxi, China; ^2^Department of Oncology, Affiliated Hospital of Youjiang Medical University for Nationalities, Guangxi, China; ^3^Department of General Surgery, Kunming Children’s Hospital, Kunming, China; ^4^Department of Pediatrics, Huai’an Second People’s Hospital, The Affiliated Huai’an Hospital of Xuzhou Medical University, Huai’an, China

**Keywords:** tumor immunotherapy, CAR-T cells, T memory cell, B-cell acute lymphocytic leukemia, tumor treatment

## Abstract

**Background:**

Cellular immunotherapies, such as chimeric antigen receptor modified-T cell (CAR-T) therapy, offers excellent potential for tumor treatment. The memory phenotype of CAR-T has been correlated positively with a therapeutic effect on and prognosis of cancer.

**Method:**

The proliferation rates of novel CAR-T was determined by cell counting. The phenotypes of CAR-T cells were then detected by flow cytometry. The cell cytotoxicity against tumor cells *in vitro* was investigated by lactate dehydrogenase assay and luciferase assay. The cytokines secreted during these assays were determined by the cytometric bead array assay. The antitumor ability *in vivo* was evaluated in NOG mice.

**Results:**

Co-expression of an LRP6 full-length protein with anti-CD19 CAR significantly improved the memory phenotype of CAR-positive T-cells by enhancing the wnt signaling pathway. As compared with anti-CD19 CAR-T, anti-CD19 CAR-T-LRP6 exhibited more robust cytotoxicity against tumor cells *in vitro* and *in vivo*, albeit fewer cytokines were released *in vitro*. Moreover, the longer survival rate and robust expansion *in vivo* of anti-CD19 CAR-T-LRP6 cells were found to be effective in inhibiting cancer recurrence.

**Conclusions:**

CAR co-expressed with LRP6 could sustain the memory phenotype that enabled permanent relief and may further assist in the development of potent and durable T-cell therapeutics.

## Introduction

Recently, chimeric antigen receptor-engineered T (CAR-T) cell therapy was suggested as one of the most promising approaches for tumor treatment, especially for leukemia and lymphoma (1). CD19-specific CAR has induced remarkable responses in patients with refractory leukemia and lymphoma (1, 2). Two FDA-approved CAR-T-cell therapies targeting the CD19 molecule are available: the tisagenlecleucel (3–5) for adult B-cell acute lymphoblastic leukemia (B-ALL) and the axicabtagene ciloleucel for large B-cell non-Hodgkin lymphoma (6, 7). Although the efficacy of CAR-T therapy is unprecedented, the treatment of relapse after CAR-T therapy remains challenging (8).

One of the reasons for relapse after CAR-T therapy is the short lifetime of CAR-T cells that make them difficult to detect in the peripheral blood, which may also explain the immunological rejection or exhaustion (9, 10). Throughout the lifespan of a T cell, the central memory T cells, which express the homing receptors CCR7 and CD62L, reside in the lymph nodes, awaiting stimulatory signals from the antigen-presenting cells (APC). On stimulation, the central memory T cells begin to proliferate and differentiate into effector T cells and then egress to the lymph nodes. On stimulation by the target cells, these effector T cells exhibit a range of robust cytolytic functions, including apoptosis (11, 12). Several clinical studies have demonstrated that the clinical response to anti-CD19 CAR-T is positively correlated with an increasing population of the central memory T cells (13).

Many studies have shown that the wnt signaling pathway is beneficial for the persistence of memory T cells and for the inhibition of differentiation between memory and effector T cells (14–17). After the Wnt ligand binds to receptors such as LRP and FZD, the adaptor and downstream proteins get phosphorylated, which in turn promotes the activation of β-catenin for interaction with numerous DNA binding proteins in order to remodel the chromatin and orchestrate the transcriptional programs (18, 19). Brennan et al. demonstrated that the overexpression of LRP5 and LRP6 could increase the level of wnt signaling by regulating FZD in 293T cells (20). These reports suggest a new approach to optimize the phenotype of T cells by enhancing the Wnt signaling intensity.

In the current research, we designed a novel CAR co-expressing full-length LRP6 with anti-CD19 CAR on the surface of T cells (α19BBZ-LRP6). Our experiments revealed that the overexpression of LRP6 could promote the proliferation of memory T cells and sustain the memory phenotype *ex vivo*. In addition, the α19BBZ-LRP6 T cells demonstrated a robust cell-killing ability against B-ALL tumor cells *in vitro* and *in vivo*. These results may provide an insight to the development of new strategies to optimize the phenotype variants and functioning of CAR-T cells for future clinical treatments.

## Materials and Methods

### Lentivirus Vector Construction and Lentivirus Package

For the construction of CAR α19BBZ, first, CAR was constituted from anti-CD19 scfv FMC63, the hinge and transmembrane domain of CD8, 4-1BB, and CD3z intracellular domain. The CAR α19BBZ-LRP6 was designed based on the structure of CAR α19BBZ co-expressing LRP6 full-length protein linked by a 2A linker. α19BBZ and α19BBZ-LRP6 were cloned into lentivirus vectors.

For preparing the lentivirus package, 293T cells were transfected with a lentivirus vector and two co-expression vectors. After 48 h, the supernatant of 293T cells was collected and concentrated by ultracentrifugation, as described elsewhere (8).

### Cell Culture

Human peripheral blood mononuclear cells (PBMCs) were purified from the peripheral blood using Ficoll reagent derived from healthy human donors and cultured in x-VIVO15 media (LONZA) supplemented with 100 IU/mL IL2 and 1% penicillin/streptomycin (P/S; Gibco, United States).

The tumor cell line Nalm6 and Raji were obtained from American Type Culture Collection (ATCC). The cell lines were cultured in 1640 media with 10% fetal bovine serum (FBS; Gibco).

### CAR-T Cell Preparation

The PBMCs were activated with CD3 and CD28 monoclonal antibodies (BioLegend) that were immobilized in a 12-well plate. After 24 h of incubation, lentivirus was added to the plate at a 10 MOI ratio for another 48 h. The cells were expanded in x-vivo 15 culture medium (Lonza) supplemented with 200 U/mL rIL-2 (R&D system).

### Flow Cytometry and Apoptosis Assay

Data were collected on the Beckman Cytoflex and analyzed by the FlowJo software. The cells (1 × 10^6^) were stained by antibodies and PE-Labeled Human CD19 protein in phosphate-buffered saline (PBS) for 30 min at 4°C. The antibodies used were PE-Labeled Human CD19 (CD9-HP254; ACRO system), anti-LRP6 (ab134146; Abcam), APC anti-human CD95 (Fas) Antibody (Biolegend 305612), and anti-CD45RA-BV421 (562885), anti-CCR7-BV510 (563449), CD4-PE CY7 (560644), and CD8-BV421 (740093) (BD Bioscience).

For the cell death assay, CAR-T cells (2 × 10^5^) and Nalm6 were co-cultured for 24 h and stained by anti-human CD3-APC antibody for 30 min. Before analysis by fluorescence-activated cell sorting (FACS), the cells were labeled with propidium iodide solution (PI; Sigma) for 5 min. The apoptosis T cells were defined as CD3+/PI+.

### Cytotoxicity Assay

Tumor cells Raji and Nalm6 (1 × 10^4^) were co-cultured with effector cells CAR-T and NT-T at a gradient ET ratio for 4 h. Next, 50 μL of the supernatant was collected, followed by detection of the lactate dehydrogenase (LDH) content. The experiment was conducted as specified by the manufacturer using the CytoTox 96^®^ Non-Radioactive Cytotoxicity Assay Kit (Promega). For the luciferase assay, Nalm6 cells (1 × 10^4^) expressing luciferase were co-cultured with CAR-T cells or NT-T cells at the 0.1 or 0.5 ET ratio for 18 h in a 96-well round-bottom culture plate (Corning 3799). After 18 h of co-culturing, the cells were centrifuged and the supernatant was discarded. The collected cells were suspended in 50 μL of PBS buffer. The cells were next transferred into a 96-well Black assay plate (Corning 3603) to which 50 μL of Dual-Luciferase^®^ Reporter Media was added, followed by incubation at room temperature for 20 min. The fluorescence was finally measured using the Envision Multilabel Reader (PerkinElmer).

The specific lysis was calculated using the following formula:

[(experimental - spontaneous release)/(maximum load - spontaneous release) x 100(%)]

(LDH assay). (1 - experimental/maximum load) x 100 (%)(luciferase-based assay).

### Cytokine Release Assay

Tumor cells (5 × 10^4^) were co-cultured with the same amount of effector cells for 18 h and then centrifuged. The cell supernatant was collected and the amount of cytokines in the supernatant was analyzed by using the cytometric bead array (CBA) Kit (560484; BD Bioscience).

### Western Blotting

Whole-cell lysates of T cells were prepared by lysing 3 × 10^6^ cells and quantified using the BCA Protein Assay Kit (C503021; Sangon Biotech). Next, 20 μg of protein was separated by electrophoresis on sodium dodecyl sulphate-polyacrylamide gel electrophoresis (SDS-PAGE) and then transferred onto polyvinylidene difluoride (PVDF) membranes and stained by anti-β-catanin (8480T; CST) and GSK-3β (12456; CST) primary antibody overnight at 4°C after blocking with 5% bovine serum albumin (BSA) and incubating in horseradish peroxidase (HRP)-linked secondary antibodies (1:1000) for 2 h at 37°C. The final signal was visualized using the enhanced chemiluminescence (ECL) detection system.

### Mouse Xenograft Studies

Xenograft studies were conducted on NOG mice (Shanghai Model Organisms Center, Inc.), with >6 mice in each group. The mice were intravenously injected with 1 × 10^6^ Nalm6 cells on day 0, and with 1 × 10^6^ CAR-T cells and NT-T cells on day 7.

At the end of every seven days of Nalm6 injection, 50 μL of the peripheral blood was collected and processed to remove the red blood cells using red cell lysis buffer (Beyotime, Shanghai, China). The T cells and Nalm6 cells were stained with anti-human CD19 and CD3 antibodies. The counts of T cells and Nalm6 cells were analyzed by the CountBright^TM^ Absolute Counting Beads (C36950, Thermo Fisher Scientific), as suggested by the manufacturer.

### Statistical Analysis

The data obtained were analyzed using the GraphPad Prism 6.0 software. One-way analysis of variance (ANOVA) and two-way ANOVA with Bonferroni post-test or T test were adopted for different conditions. *P* < 0.001, <0.01, and <0.05 suggested statistical significance.

All experiments were performed ≥3 times to ascertain reproducibility using independent donors’ cell. To ensure the correlation between the results of phenotype and functional assay, all the PBMC used in this research were collected from the same donor simultaneously and cryopreserved in liquid nitrogen, except for the case indicated in Figure 2B.

## Results

### Construction and Generation of CAR-T Cells

**FIGURE 1:**
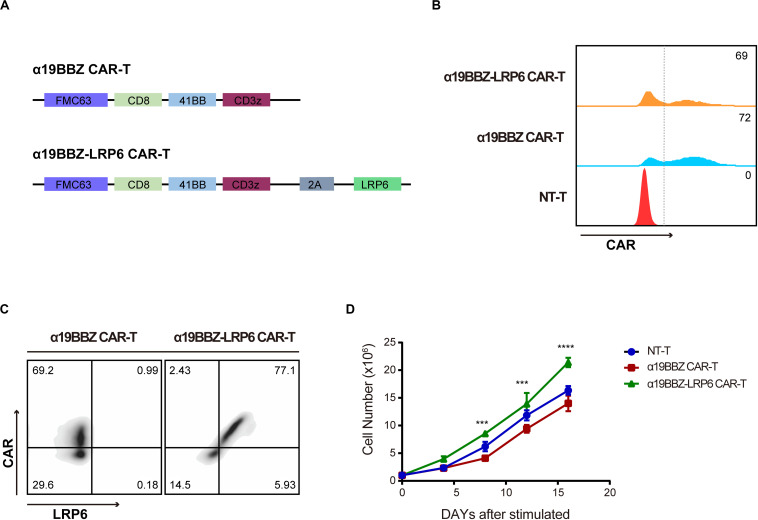
The Characteristics of CARs. **(A)** The schematic of the CAR structure showing FMC63 scfv, CD8, 41BB, and CD3z with sequential connectivity. LRP6 is linked by a 2A sequence. **(B)** The expression of CAR on T cells. **(C)** The expression of LRP6 on the surface of CAR-T cells. **(D)** The proliferation curve of CAR-T cells and NT-T cells. The data was analyzed using the GraphPad Prism 6.0 software and 2-way ANOVA. Error bars indicate mean ± SD (*n* = 3); ***P < 0.001; ****P < 0.0001

The αCD19 scFv was used to produce a second-generation CAR structure containing a 4-1BB co-stimulation domain and a CD3 zeta ITAM motif (α19BBZ). The LRP6 full-protein was co-expressed with α19BBZ linked with a 2A linker (α19BBZ-LRP6), as shown in Figure 1A. The T cells were stimulated by immobilized OKT3 and OKT28 and transfected by lentivirus, followed by the determination of the transfection efficiency of the CAR-T cells by flow cytometry after staining with PE-labeled CD19 protein (CD19-PE) (Figure 1B). To confirm whether LRP6 was expressed on the surface of α19BBZ-LRP6, the α19BBZ-LRP6 and α19BBZ T cells were examined using CD19-PE protein and anti-LRP6 antibody, followed by labeling with APC-linked anti-rabbit IgG APC antibody. As shown in Figure 1C, the expression of LRP6 was significantly upregulated on α19BBZ-LRP6 T cells when compared with that on α19BBZ. On 14-day culturing *ex vivo*, the α19BBZ-LRP6 exhibited a more robust proliferation as compared with α19BBZ; no significant difference in expression was, however, noted in comparison with those of the non-transfection T cells (NT-T group) (Figure 1D). These results demonstrate that the overexpression of LRP6 on the surface of CAR-T cells can promote T cell proliferation *ex vivo*.

**FIGURE 2:**
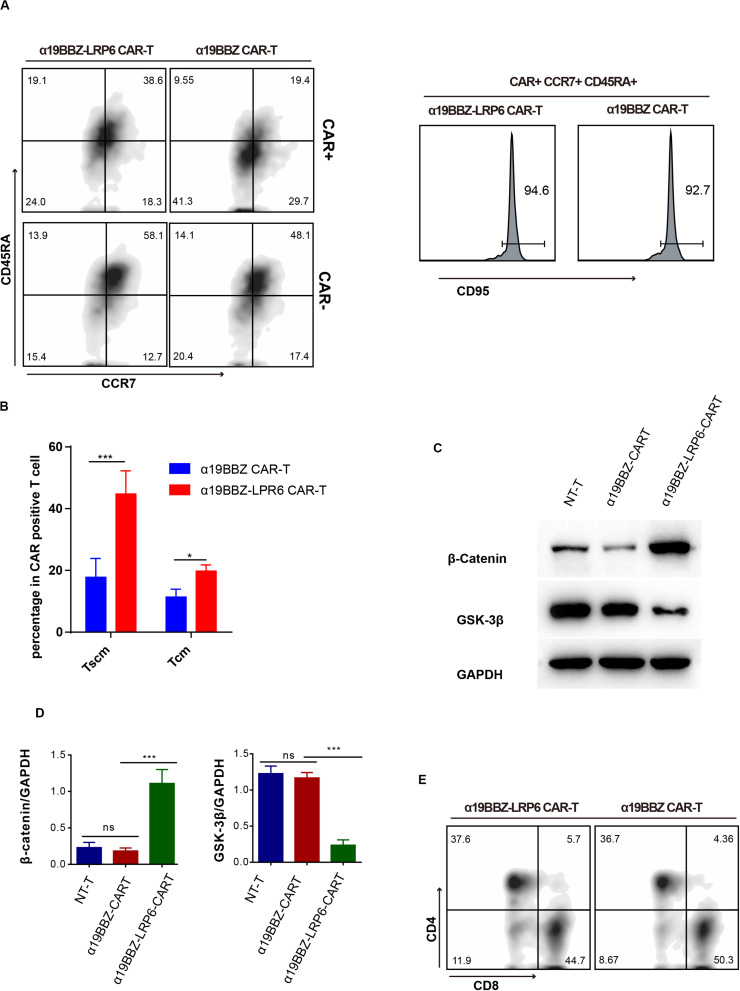
The Phenotype of CAR-T cells. **(A)** Representative flow cytometric analysis for memory biomarker detection and **(B)** statistics of Tscm from five donors. **(C)** Flow cytometry for CD4 and CD8 proportion. **(D)** Western blotting and statistical analyses for the wnt signaling pathway in CAR-T cells and NT-T cells. Error bars indicate mean ± SD (*n* = 3), *P < 0.05; **P < 0.01; ***P < 0.001.

### The Phenotype of α19BBZ-LRP6 CAR-T Cells

In order to confirm whether the overexpressed LRP6 on the CAR-T cells could promote the lifespan of memory T cells, the CAR-T cells were labeled with anti-CD45RA-BV421, anti-CCR7-BV510, anti-CD95-APC, and CD19-PE. As per past studies, Tscm and Tcm can be defined as CD45RA+CCR7+CD95+ and CD45RA-CCR7+ (21, 22). As shown in Figure 2A, in comparison with α19BBZ T cells, the α19BBZ-LRP6 cells displayed a greater proportion of TCM (CD45RA-CCR7+) and TSCM (CD45RA+CCR7+CD95+) in CAR-positive cells. In addition, it is noteworthy that the CAR-positive T cell population showed greater TCM and TSCM proportions than the CAR-negative population in α19BBZ-LRP6 CAR-T cells, but not in α19BBZ CAR-T cells. To exclude individual differences, this experiment was repeated five times using PBMC samples from different donors; the results were found to be similar throughout (Figure 2B), confirming that overexpressed LRP6 could optimize CAR-T by maintaining the memory phenotype population of T cells. Reportedly, the co-overexpression of LRP6 can stabilize β-catenin and enhance the Wnt signaling pathway *in vitro* (20). In consideration that the Wnt pathway could play an important role in T cell differentiation, we examined whether overexpressed LRP6 could enhance the Wnt signaling pathway in CAR-T cells (Figures 2C,D). The amounts of GSK-3β and stabilized β-catenin were analyzed by western blotting. As expected, the level of stabilized β-catenin in α19BBZ-LRP6 CAR-T cells were >5-fold greater than that in α19BBZ CAR-T cells. The content of GSK-3β in α19BBZ-LRP6 was significantly lower than that in NT-T and α19BBZ T cells. These results indicate that overexpression of LRP6 on the surface of CAR-T cells could upregulate the wnt-β-catenin signaling pathway. However, no significant difference was noted in the CD4/CD8 ratio between α19BBZ-LRP6 and α19BBZ T cells. This result demonstrates that the function of overexpressed LRP6 on CD4 and CD8 T cells is similar (Figure 2E). In total, we found that the overexpression of LRP6 with anti-CD19 CAR could facilitate the sustenance of memory phenotypes with the enhancement of the Wnt signaling pathway.

### The Anti-tumor Capability of α19BBZ-LRP6 CAR-T Cells *in vitro*

**FIGURE 3:**
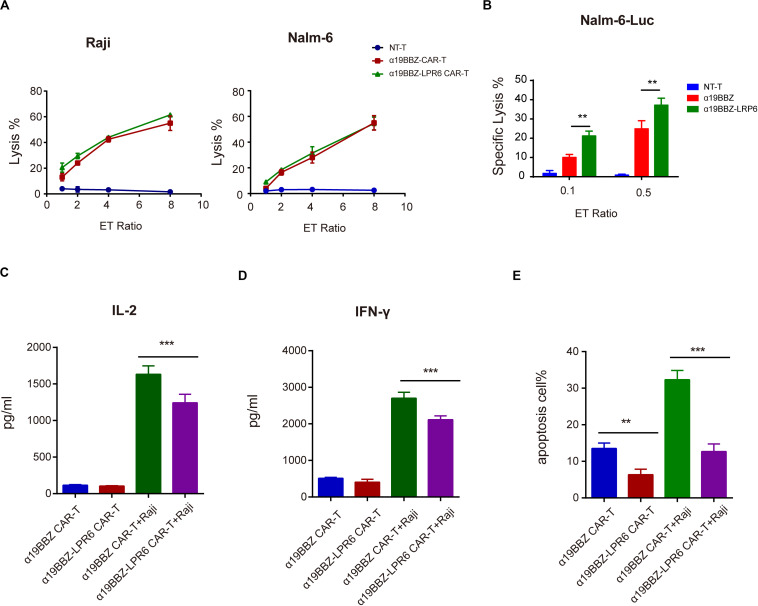
Function of CAR-T to tumor cells. Specific lysis for **(A)** Raji and **(B)** Nalm6 for 4 h and against Nalm6-Luc for 18 h. **(C)** IL-2 and **(D)** IFN-gamma released. **(E)** The apoptosis of CAR-T cells after stimulation by tumor cells. Error bars indicate mean ± SD (*n* = 3), *P < 0.05; **P < 0.01; ***P < 0.001.

To determine the short-term cytotoxicity of CAR-T cells targeting CD19-positive tumor cells, Raji and Nalm6 were co-cultured with α19BBZ-LRP6 CAR-T cells, α19BBZ CAR-T cells, and NT-T cells for 4 h at a gradient ET ratio. The rate of lysis of tumor cells is illustrated in Figure 3A. Strong lysis for both Raji and Nalm6 was recorded in α19BBZ-LRP6 CAR-T cells and α19BBZ CAR-T cells, but not in NT-T cells. The cytotoxicity of α19BBZ CAR-T cells and α19BBZ-LRP6 CAR-T cells showed no significant difference. For the long-term activity of anti-tumor detection, the luciferase-expressing tumor cell Nalm6-Luc was co-cultured with CAR-T cells or NT-T cells at 0.1 or 0.5 ET ratio for 18 h. As shown in Figure 3B, the α19BBZ-LRP6 CAR-T cells exhibited a stronger ability of tumor lysis in comparison with α19BBZ CAR-T cells. To determine the level of related cytokines secreted, the supernatant of the co-cultured CAR-T cells and tumor cells were collected and evaluated by using the CBA Kit. Increased release of IFN-γ and IL-2 could be detected in the supernatants of both α19BBZ-LRP6 CAR-T cells and α19BBZ CAR-T cells in comparison with that in NT-T cells. In addition, the amounts of IFN-γ and IL-2 were lower in α19BBZ-LRP6 CAR-T cells than that in α19BBZ CAR-T cells after stimulation by the tumor cells (Figures 3C,D), which suggests that the α19BBZ-LRP6 T cells may have a lower risk of developing cytokine-releasing syndrome. After 24 h of co-culturing with Nalm6 cells, the number of apoptotic cells in the α19BBZ-LRP6 group was significantly lower than that in the α19BBZ CAR-T cells (Figure 3E). Taken together, the α19BBZ-LRP6 CAR-T cells may have a similar capacity of tumor cell lysis, while the overexpression of LRP6 with anti-CD19 CAR exhibited a greater memory T cell population and lower apoptosis of the T cell population after stimulation.

### Antitumor Effect of α19BBZ-LRP6 CAR-T Cells *in vivo*

**FIGURE 4:**
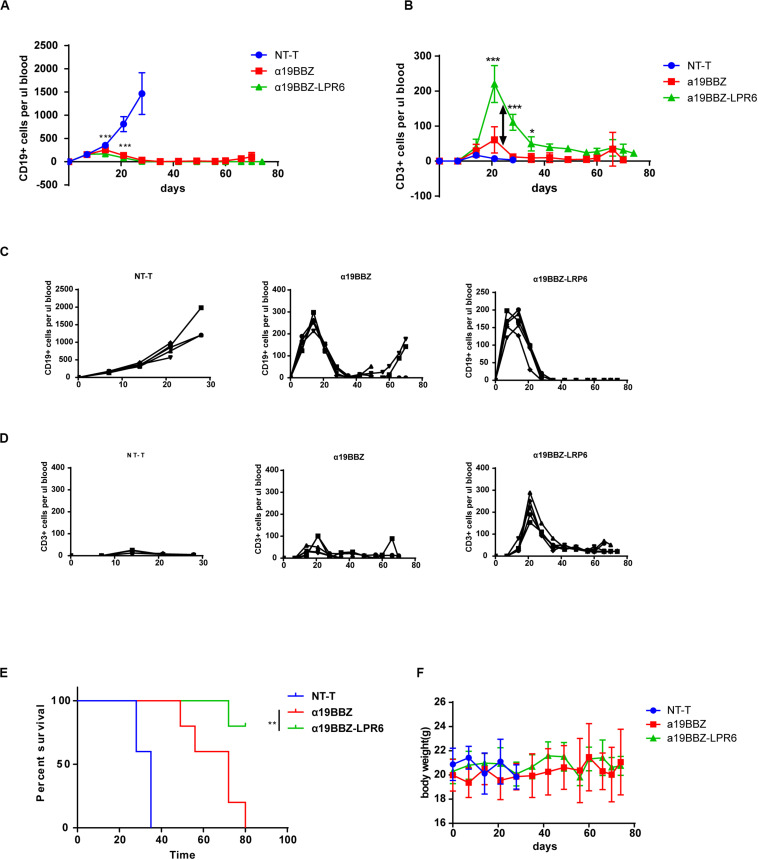
Antitumor efficacy of CAR-T cells in Nalm6 xenograft model. **(A,B)** Representation of the human CD19-positive tumor cells surviving in the peripheral blood. **(C,D)** Representation of the human CD3 + T cells in the peripheral blood. **(E)** Representation of the mice survival curve calculated using the Kaplan-Meier method. **(F)** The curve of body weight of mice. Error bars indicate mean ± SD (*n* = 5), *P < 0.05; **P < 0.01; ***P < 0.001; ****P < 0.0001.

In order to determine the antitumor effect of novel CAR-T cells *in vivo*, we established an animal model using the Nalm6 Xenografts in NOG mice. Nalm6 cells (1 × 10^6^) were intravenously injected into the experimental mice seven days after Nalm6 injection; 50 μL of the peripheral blood was collected from the orbit every 3–4 days, and the amount of human CD19-positive cells in the blood was estimated. When the density of CD19-positive cells was >50/μL, 1 × 10^6^ CAR-T cells or normal T cells were injected into the tail vein on day 7. Then, the density of human CD3+ and CD19+ cells in the peripheral blood were counted every seven days. As per the results, the α19BBZ-LRP6 CAR-T cell group demonstrated a better anti-tumor response for the proliferation of human CD3+ T cells than the α19BBZ CAR-T group (Figures 4A–D). In addition, prolonged survival was recorded for the CAR-T treatment groups, both for α19BBZ and α19BBZ-LRP6 CAR-T cell groups, but not for the NT-T cell group (Figure 4E). Our cumulative results thus illustrated that the α19BBZ-LRP6 CAR-T cells possess a high-potential therapeutic efficacy for B-ALL.

## Discussion

**FIGURE 5:**
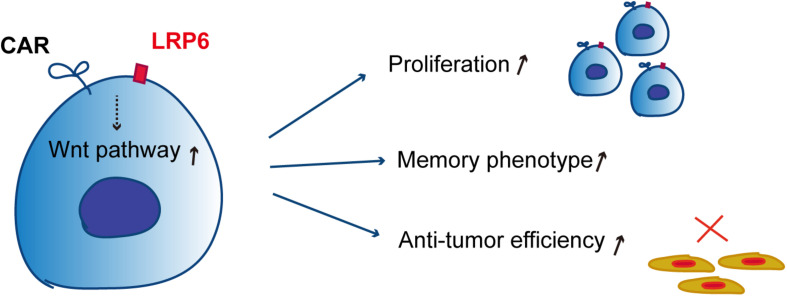
Schematic of the mechanism of co-expression of LRP6 and CAR-T. The co-expression of LRP6 with α19BBZ could sustain the memory phenotype and exhibit a greater ability of proliferation and anti-tumor capacity *in vitro* and *in vivo* by enhancing the wnt-signaling pathway.

In our recent past research, LRP6 was overexpressed for the first time in CAR-T therapy for sustaining memory T cells. In the present report, overexpressed LRP6 upregulated the population of TSCM and TCM by enhancing the Wnt signaling pathway during culture *ex vivo*, which may have contributed to the CAR-T cell proliferation and cytotoxicity both *in vitro* and *in vivo* (Figure 5).

Joseph et al. enriched CAR-T cells from patients with a good clinical response in gene expression profiles involved in early memory differentiation, such as in the expression of TCF7 and LEF1 (13). The memory T cells showed weaker cytotoxicity than effector T cells, but the memory T cells presented with a tendency of differentiation and proliferation, rather than apoptosis like that by effector T cells, on stimulation (12). Several studies have indicated that the infusion of less-differentiated T cells could result in greater cell expansion, persistence, and tumor destruction (23). The persistence of memory CAR-T cells for a long time facilitates the monitoring and killing of tumor cells at any time to prevent recurrence.

IL7, IL15, or IL21 have been reported to be involved in the maintenance of memory T cells during culture *ex vivo* (21, 24–26). These cytokines play a significant role in stabilizing the metabolic profiles of T cells and in sustaining the memory T cell population; however, the memory phenotype *in vivo* may not be maintained due to the lack of an environment of abundant cytokines. In our recent research, LRP6 overexpressed on the surface of CAR-T cells was found to benefit self-renewal and the sustenance of memory CAR-T cells for the promotion of proliferation and cytotoxicity *in vivo* and *in vitro*.

Several studies have demonstrated the benefits of memory phenotype maintenance of T cells toward activating the Wnt signaling pathway (15, 17). For instance, Sabatino reported that clinical grade CAR modified TSCM could be efficiently induced by IL-7, IL-21, and the glycogen synthase-3β (GSK-3β) inhibitor TWS119 (22). However, the demerit of this approach is that the addition of cytokines and the small molecule inhibitors targeting the Wnt signaling pathway would increase the cost of preparation and quality control of CAR-T cells. In this study, LRP6, the essential component for memory maintenance, was co-expressed with CAR in the same open reading frame (ORF), which helped retain the cost in comparison with that for the “conventional CAR.”

## Conclusion

In summary, we have described a novel anti-CD19 CAR co-expressing the Wnt receptor LRP6, which exhibited a significant capacity for sustaining and renewing the memory phenotype of T cells. Moreover, we demonstrated that α19BBZ-LRP6 CAR-T has more potent cytolytic activity and a longer survival duration *in vitro*. Furthermore, we found that the co-expression of LRP6 could prolong the survival of tumor-bearing mice *in vivo*, which suggests its potential to develop a promising strategy for clinical treatment of cancer in the future.

## Data Availability Statement

The raw data supporting the conclusions of this article will be made available by the authors, without undue reservation.

## Ethics Statement

The studies involving human participants were reviewed and approved by Ethics Committee of the Xuzhou Medical University. Written informed consent for participation was not required for this study in accordance with the national legislation and the institutional requirements. The animal study was reviewed and approved by Ethics Committee of the Xuzhou Medical University.

## Author Contributions

C-LW and S-SY designed the study and performed the experiment. C-LW and S-SY collected the data and made the analysis. S-SY wrote the manuscript draft. All authors approved the final version of the manuscript.

## Conflict of Interest

The authors declare that the research was conducted in the absence of any commercial or financial relationships that could be construed as a potential conflict of interest.

## Abbreviations

APC: antigen-presenting cell
B-ALL: B-cell acute lymphoblastic leukemia
CAR-T: chimeric antigen receptor modified T cell.
